# Microbial production of rhamnolipids: opportunities, challenges and strategies

**DOI:** 10.1186/s12934-017-0753-2

**Published:** 2017-08-05

**Authors:** Huiqing Chong, Qingxin Li

**Affiliations:** Institute of Chemical and Engineering Sciences, Agency for Science, Technology and Research, 1 Pesek Road, Jurong Island, 627833 Singapore

**Keywords:** Rhamnolipids, *Pseudomonas,* fermentation, Metabolic engineering, Application

## Abstract

Rhamnolipids are a class of biosurfactants which contain rhamnose as the sugar moiety linked to β-hydroxylated fatty acid chains. Rhamnolipids can be widely applied in many industries including petroleum, food, agriculture and bioremediation etc. *Pseudomonas aeruginosa* is still the most competent producer of rhamnolipids, but its pathogenicity may cause safety and health concerns during large-scale production and applications. Therefore, extensive studies have been carried out to explore safe and economical methods to produce rhamnolipids. Various metabolic engineering efforts have also been applied to either *P. aeruginosa* for improving its rhamnolipid production and diminishing its pathogenicity, or to other non-pathogenic strains by introducing the key genes for safe production of rhamnolipids. The three key enzymes for rhamnolipid biosynthesis, RhlA, RhlB and RhlC, are found almost exclusively in *Pseudomonas* sp. and *Burkholderia* sp., but have been successfully expressed in several non-pathogenic host bacteria to produce rhamnolipids in large scales. The composition of mono- and di-rhamnolipids can also be modified through altering the expression levels of RhlB and RhlC. In addition, cell-free rhamnolipid synthesis by using the key enzymes and precursors from non-pathogenic sources is thought to not only eliminate pathogenic effects and simplify the downstream purification processes, but also to circumvent the complexity of quorum sensing system that regulates rhamnolipid biosynthesis. The pathogenicity of *P. aeruginosa* can also be reduced or eliminated through in vivo or in vitro enzymatic degradation of the toxins such as pyocyanin during rhamnolipid production. The rhamnolipid production cost can also be significantly reduced if rhamnolipid purification step can be bypassed, such as utilizing the fermentation broth or the rhamnolipid-producing strains directly in the industrial applications of rhamnolipids.

## Background

Surfactants are amphipathic molecules with surface activities to reduce surface tensions. They are widely utilized as detergents, solubilizers or emulsifying agents in many industrial fields such as petroleum, food, pharmaceutical and agricultural industries [1, 2]. The total worldwide production of surfactants is estimated to be over 15 million tons per year, and expected to increase to over 24 million tons annually by 2020 [3]. Currently, surfactants are mainly derived from petroleum products chemically due to the low cost of production. However, synthetic surfactants are non-biodegradable, which may lead to environmental problems. Therefore, biosurfactants produced by microbial fermentation can be used to replace synthetic surfactants as environmental friendly alternatives [4–6]. The most attractive characteristic of biosurfactant is that they are easily biodegradable and cause less toxic impact to the environment, while having similar properties to synthetic surfactants. Some biosurfactants are also tolerant to a wide range of extreme conditions (low and high pH, high temperature and high salinity) that are frequently encountered in industrial processes, as well as several interesting biological properties like antimicrobial activity [2, 7]. Biosurfactants can be classified into several categories including glycolipids (rhamnolipids, sophorolipids, trehalose lipids), lipopeptide (surfactin, iturin) and polymeric compounds (emulsan, alasan) [9]. Among them, rhamnolipids are the most extensively studied because of their excellent physicochemical properties and ability to reach high fermentation titers. Here we focus on reviewing the challenges and opportunities in microbial production of rhamnolipids, as well as potential strategies to address these challenges.

## Rhamnolipids

**Fig. 1:**
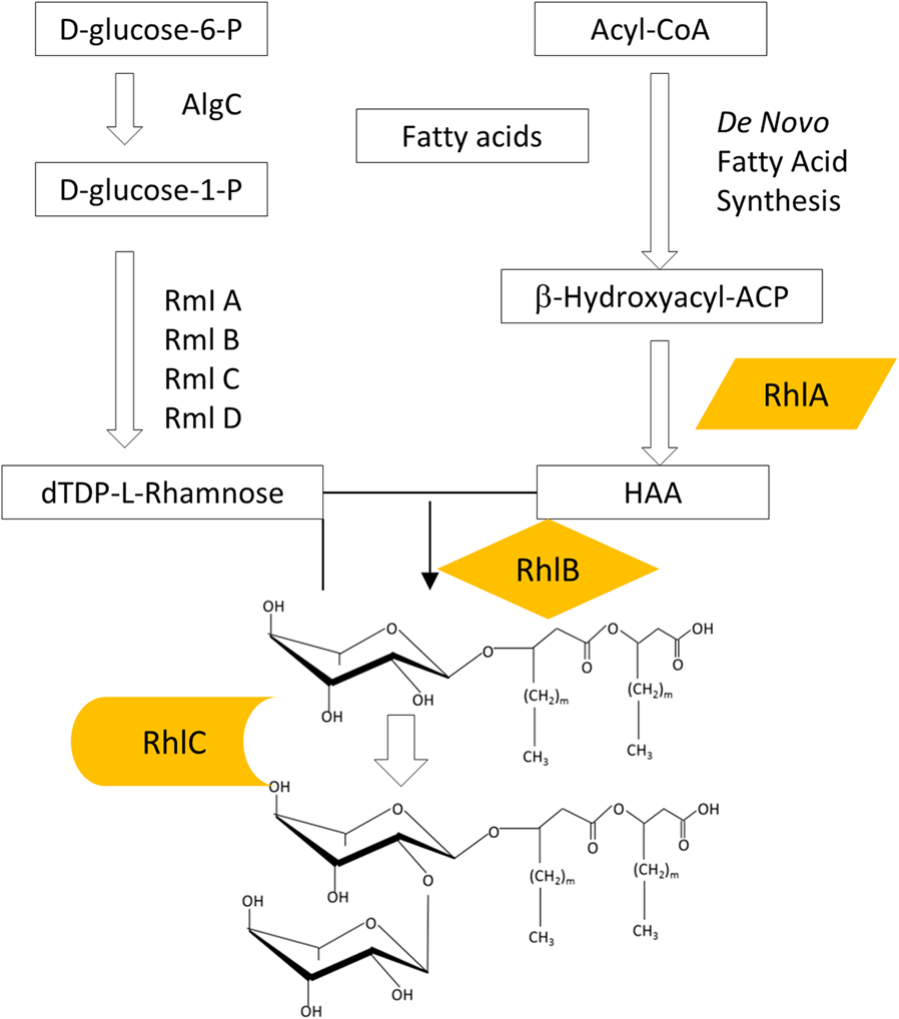
Rhamnolipid production in *Pseudomonas*. The synthesis pathway for rhamnolipids is indicated while the details for metabolic pathway of glucose and synthesis of Acyl-CoA are not provided. RhlA, RhlB and RhlC are three enzymes essential for rhamnolipid synthesis

Rhamnolipids are glycolipids containing a hydrophilic group, consisting of either one or two (l)-rhamnose molecules, with a glycosidic linkage to the hydrophobic group made up of one or two β-hydroxy fatty acids (Fig. 1). Microbial fermentation produces a diversity of rhamnolipid congeners with variations in the chain length, degree of unsaturation for the fatty acid chains, and differences in the number of rhamnose molecules. Rhamnolipids containing one and two rhamnose molecules are known as mono-rhamnolipids and di-rhamnolipids, respectively (Fig. 1). It is estimated that approximately 60 rhamnolipid congeners and homologues exist in the fermentation broth, while the predominant rhamnolipid species and the concentrations of the congeners are dependent on the rhamnolipid-producing strains [8]. The physicochemical properties of the rhamnolipids can be greatly affected even with small changes in the composition of the congeners. For instance, the critical micelle concentration (CMC) is about 230 mg/l for rhamnolipid mixture with high proportion of congeners containing unsaturated fatty acids [9], 5 mg/l for di-rhamnolipids with C_10_ fatty acids, and 40 mg/l for mono-rhamnolipids with C_10_ fatty acids [2, 10].

### Rhamnolipid applications

Rhamnolipids can be widely applied to many industrial fields such as petroleum, bioremediation, agriculture, cosmetics, food processing and pharmaceuticals because of their excellent surface activities and biological activities [11, 12]. Particularly, rhamnolipids can be used in manufacturing fine chemicals and surface coatings, as food additives and as biological control agents [6]. The applications have been described in detail in several reviews [13] and are summarized in Table 1.

**Table 1:** Applications of rhamnolipids

Applications	Examples	References
Oil recovery	Microbial enhanced oil recovery (MEOR) Increase amount of recoverable oil aided by rhamnolipid-producing microorganisms	[73] [2, 74]
Bioremediation	Bioremediation of petroleum at contaminated sites Addition of rhamnolipids improves solubility of hydrocarbons to facilitate degradation	[46, 75–78]
	Bioremediation of heavy metal at contaminated lands or in water treatment plant; rhamnolipids can be applied in foaming-surfactant technology to remove heavy metal contaminants	[2, 11, 79]
	Bioremediation of pesticides at agricultural fields; addition of rhamnolipids can enhance degradation of chemical pesticides	[11, 79]
Pest control	As emulsifier, spreaders and dispersing agent in pesticide formulations	[2, 79]
	As bio-pesticide against agricultural pests; rhamnolipids have insecticidal activity against green peach aphids and *Aedes aegypti* larvae	[43, 80]
Crop protection	As biocontrol agent against several phytopathogenic fungi; addition of rhamnolipids or rhamnolipid-containing cell-free broth are effective in inhibiting growth of phytopathogens, e.g. *F. oxysporum*, *B. cinerea*, *Mucor* spp. and many more	[1, 7, 81–84]
	As stimulant for plant immunity; induced genes involved in plant’s defense system in tobacco, wheat and *Arabidopsis thaliana*; induced biosynthesis of plant hormones important for signaling pathways involved in plant immunity	[43]
Food processing	As food ingredients or additives functioning as emulsifier, solubilizer, foaming and wetting agent	[7]
	As antimicrobial agent preventing food spoilage and for sanitization; rhamnolipids inhibit growth of foodborne pathogenic bacteria, e.g. *L. monocytogenes*, *B. subtilis*; rhamnolipids also prevent formation of biofilms due to their anti-adhesive nature	[1, 85, 86]
	As a source of (l)-rhamnose; enzymatic hydrolysis of rhamnolipids to obtain (l)-rhamnose, a raw material for production of flavor compounds	[42, 87]
Medical use	As biofilm control agent to prevent medical device-related infections; inhibit biofilm formation; synergistic effect with caprylic acid to inhibit biofilms of more resistant pathogens, e.g. *P. aeruginosa* and *S. aureus*	[2, 88, 89]
	As anticancer agent; rhamnolipids inhibit growth of many human cancer cell lines, e.g. HI-60, BV-173, SKW-3, JMSU-1 and Hela cells	[2, 19]
Protein folding	Aid in folding of outer membrane protein A	[90]
Microbial fuel cells	Improve power density output of microbial fuel cells	[91]
Synthesis of nanoparticles	As structure-directing agent in nanoparticle synthesis	[92]

### Rhamnolipid-producing strains

Rhamnolipids are thought to be produced by hydrocarbon-degrading microorganisms as the amphipathic characteristics of rhamnolipids are essential for bacteria to uptake and utilize the hydrophobic hydrocarbons as the carbon source [14]. Some microorganisms produce rhamnolipids only when hydrocarbons are used as the carbon source [15]. Some rhamnolipid-producing strains can cause nosocomial infections, especially in individuals with reduced immunity because rhamnolipids may be important for the pathogenic effects [5]. Quite a few rhamnolipid-producing strains have been isolated and characterized (Table 2). Among the available strains, the rhamnolipid yield of *P. aeruginosa* is still the highest.

**Table 2:** Isolated rhamnolipid-producing strains

Strain	Carbon source	Maximum yield (g/l)	References
*Pseudomonas aeruginosa* PAO1	2.5% (w/v) sunflower oil	36.7	[93]
*Pseudomonas aeruginosa* Z41	3% (v/v) waste frying oil	9	[94]
*Pseudomonas aeruginosa* ATCC 9027	0.5% (w/v) glucose	13.3–46.8 (rhamnose equivalents)	[95]
*Pseudomonas aeruginosa* O-2-2	8% (w/v) soybean oil + 2, 4 and 4% (w/v) after 24 h, 48 h and 72 h respectively	70.56	[65]
*Pseudomonas chlororaphis* NRRL B-30761	2% (w/v) glucose	1	[96]
*Pseudomonas fluorescens* HW-6	1.5% (w/v) hexadecane	1.4–2	[97]
*Pseudomonas fluorescens* Migula 1895	2% (v/v) olive oil	2	[98]
*Pseudomonas indica* MTCC 3714	4% (w/v) combination of rice bran, de-oiled rice bran and glucose	9.6	[99]
*Pseudomonas luteola* B17	5% (*w/v*) molasses	0.53	[100]
*Pseudomonas nitroreducens*	4% (w/v) glucose	5.46	[101]
*Pseudomonas putida* B12	5% (w/v) molasses	0.52	[100]
*Pseudomonas putida* 21BN	2% (w/v) glucose	1.2 (rhamnose equivalents)	[102]
*Pseudomonas putida* BD2	2% (w/v) glucose	0.15	[103]
*Pseudomonas stutzeri*	1% (w/v) mannitol + 1% (w/v) coal	8.74 (rhamnose equivalents)	[104]
*Burkholderia glumae*	2% (w/v) canola oil	1.007	[105]
*Burkholderia kururiensis* KP23^T^	3% (w/v) glycerol	0.78	[48]
*Burkholderia plantarii* DSM 9509^T^	1% (w/v) glucose	0.04574	[106]
*Burkholderia pseudomallei* NCTC 10274	4% (v/v) glycerol	NA	[107]
*Burkholderia thailandensis* E264	4% (v/v) canola oil	1.473	[108]
	4% (v/v) glycerol	2.79	[109]
*Acinetobacter* sp. YC-X 2	0.186% (w/v) *n*-hexadecane	1.15	[110]
*Acinetobacter calcoaceticus* NRRL B-59190	1% (v/v) glycerol	2	[12, 111]
	2% (w/v) sodium citrate	1.2	[12]
*Acinetobacter calcoaceticus* NRRL B-59191	1% (v/v) glycerol	2.2	[111]
*Enterobacter asburiae* NRRL B-59189	1% (v/v) glycerol	2	[111]
	2% (w/v) sodium citrate	0.51	[12]
*Enterobacter hormaechei* NRRL B-59185	1% (v/v) glycerol	2.4	[111]
*Nocardioides* sp. A-8	2% (w/v) *n*-paraffin	NA	[112]
*Pantoea stewartii*	1% (v/v) glycerol	2.2	[111]
*Pseudoxanthomonas* sp. PNK-04	2% (w/v) mannitol	0.28	[113]
*Renibacterium salmoninarum* 27BN	2% (w/v) *n*-hexadecane	0.8 (rhamnose equivalents)	[114]
*Serriatia rubidaea* SNAU02	2.931% (w/v) mannitol	NA	[33]
*Streptomyces* sp. ISP2-49E	0.4% (w/v) glucose	NA	[115]
*Tetragenococcus koreensis* sp. nov.	1% (w/v) glucose + 1% (w/v) sodium acetate	NA	[116]
*Thermus aquaticus* CCM 3488	0.2% (w/v) sunflower oil	2.79	[117]
*Thermus* sp. CCM 4212	0.2% (w/v) sunflower oil	2.12	[117]
*Meiothermus ruber* CCM 2842	0.2% (w/v) sunflower oil	1.505	[117]

The non-reported yield is labeled as NA

## Rhamnolipid synthesis and regulation in *P. aeruginosa*

*Pseudomonas aeruginosa* has been used as the model strain to understand the genes that are critical for rhamnolipid biosynthesis. Production of rhamnolipids in *Pseudomonas* involves several steps. The precursors for rhamnolipid synthesis are the sugar (dTDP-l-rhamnose) and hydrophobic moieties such as 3-(3-hydroxyalkanoyloxy)alkanoic acid (HAA). The sugar moiety can be synthesized from d-glucose, while the hydrophobic moiety can be synthesized through the fatty acid synthesis pathway, starting with two-carbon units [16]. Most bacteria contain the required enzymes for synthesizing the precursors in rhamnolipid biosynthesis but the enzymes involved in the synthesis of HAA, mono- and di-rhamnolipids are found almost exclusively in *Pseudomonas* sp. and *Burkholderia* sp. (Fig. 1). Synthesis of rhamnolipids and their precursors only occurs upon induction of the related gene products to express the key enzymes for the rhamnolipid biosynthesis pathway.

### Quorum sensing system

Bacterial quorum sensing (QS) is bacterial communication system where signal molecules known as auto-inducers are secreted to generate coordinated behaviors within a bacterial population. The QS system consists of a signal synthase, a signal receptor protein and a signal molecule in many gram-negative bacteria [4]. In *P. aeruginosa*, there are three QS systems including *las*, *rhl* and *pqs* systems that are responsible for regulating rhamnolipid production [4, 6, 17–19]. The LasI and RhlI synthases produce corresponding signal molecules, homoserine lactones 3OC_12_-HSL and C_4_-HSL, which bind and modulate LasR and RhlR modulators, respectively. RsaL can affect rhamnolipid biosynthesis by suppressing both LasI and LasR [4]. The *rhl* system directly controls the biosynthesis of rhamnolipids through the transcription factor RhlR [20] and its autoinducer C_4_-HSL. C_4_-HSL forms a complex with RhlR to activate the transcription of *rhlAB* genes to initiate rhamnolipid biosynthesis during stationary phase [20–22]. It has been shown that 1 µM RhlI or 10% of cell-free supernatant is sufficient for full induction of rhamnolipid biosynthesis, which usually starts 3 h after induction. However, the maximal production of rhamnolipids only occurred 20–36 h after induction [20]. In contrast, exogenous addition of C_4_-HSL to the culture medium is not helpful for initiating the expression of *rhlAB* during exponential phase, which implies that additional regulatory elements are present to prevent the initiation of rhamnolipid biosynthesis before the onset of stationary phase [23]. Although the addition of exogenous auto-inducer did not boost rhamnolipid production, it was found that the presence of 20% spent medium helped to increase rhamnolipid yield by 9.39%, which might be due to the presence of endogenous auto-inducer in the spent medium [24]. One of the functions of rhamnolipids is to increase the bioavailability of hydrocarbon substrate as carbon source [15]. Hence, the synthesis of rhamnolipids can also be induced by the presence of water-insoluble substrates in the fermentation medium such as hydrocarbons [25, 26], which has been used as a strategy to improve rhamnolipid production.

### Key enzymes in rhamnolipid synthesis

The lipid moiety of rhamnolipid is synthesized by the classical type-II fatty acid synthase (FAS II) which exists in most bacteria. The formation of the precursor HAA that constitute the hydrophobic portion of rhamnolipids is catalyzed by RhlA, using β-hydroxydecanoyl-ACP from the fatty acid synthesis pathway as the substrate. Rhamnose can be found in the lipopolysaccharide (LPS) component of cell wall in *Pseudomonas* species and O-antigen polysaccharides of some gram-negative bacteria. The precursor dTDP-L-rhamnose for rhamnolipid biosynthesis is synthesized from d-glucose-1-phosphate, which can be obtained from both gluconeogenesis and Entner–Doudoroff pathways utilizing d-glucose as the starting molecule [4]. Phosphoglucomutase AlgC and gene products of *rmlBDAC* operon are essential for producing dTDP-l-rhamnose from d-glucose-1-phosphate [27]. Most of the enzymes involving in rhamnolipid biosynthesis are present in many bacteria except for the three key enzymes RhlA, RhlB and RhlC.

As aforementioned, RhlA catalyzes formation of HAA—the hydrophobic precursor of rhamnolipids. RhlB is a rhamnosyltransferase that is capable of catalyzing the reaction between HAA and dTDP-l-rhamnose to form mono-rhamnolipid. RhlA and RhlB are encoded by the *rhlAB* operon that is tightly controlled by the QS systems (Fig. 1). RhlC, encoded by the *rhlC*, is a rhamnosyltransferase II that is responsible for production of di-rhamnolipids using mono-rhamnolipid and another dTDP-l-rhamnose as substrates. RhlA, RhlB and RhlC, which are important enzymes to control the production of rhamnolipids, are found almost exclusively in *Pseudomonas* sp. and *Burkholderia* sp. Theoretically, non-pathogenic strains will be able to produce rhamnolipids when these three genes are introduced. The population of mono- and di-rhamnolipids can also be altered by varying the expression levels of RhlB and RhlC. Indeed, many studies have been carried out to obtain rhamnolipid-producing strains through metabolic engineering [4]. The recombinant bacteria harboring these genes are able to produce rhamnolipids (see below).

## Challenges in microbial production of rhamnolipids

Several laboratory studies have proven the possibility of producing rhamnolipids in pilot scale, and companies such as Evonik are known to have spent efforts in exploring large scale production of rhamnolipids for commercialization [28, 29]. Despite these progresses, production of biosurfactants by microbial fermentation can only replace synthetic surfactants when the cost of raw materials and process is reduced [29]. The high cost for rhamnolipid production is mainly due to fermentation and product purification steps. The cost of synthetic surfactants is $1–3/kg, whereas rhamnolipids cost $20–25/kg depending on the volumetric productivity of rhamnolipid fermentation [30, 31]. Due to higher production cost in fermentation, it is difficult for rhamnolipids to compete with synthetic surfactants economically. Microbial fermentation to produce rhamnolipids is only economically viable when rhamnolipids are required in the manufacturing of high-priced products such as cosmetics and medicines [32]. Nonetheless, as the demand for surfactants is increasing annually, it is still important to explore strategies to reduce the cost for rhamnolipids production [30].

**Fig. 2:**
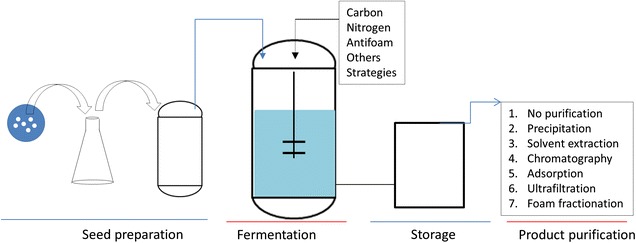
A flow chart of rhamnolipids production. This flow chart shows the procedures for rhamnolipid production. A large scale rhamnolipid production contains the following steps: seed preparation, fermentation, production purification. For a given strain, the costs are focused on the fermentation and purification steps. Seed preparation for fermentation is a common step which does not require extra cost. Fermentation cost includes carbon and nitrogen sources, fermentation styles, and other additional steps or chemicals required for rhamnolipid production. Cost for production purification depends on the required production purity. Some applications may not require rhamnolipid purification

The costs involved in rhamnolipid production originate from the raw materials to serve as carbon and nitrogen sources for the microorganism, the fermentation procedures and the downstream processes (Fig. 2). The cost for carbon source is high because hydrophobic substrates are usually used to achieve high product yield, and such substrates are normally more expensive than sugar-based substrates. The cost of raw materials for rhamnolipid fermentation was estimated to be 50% of the total production cost [30]. Fermentation strategy is also important for saving operation cost and increasing the product titers. Quite a few studies have shown that fermentation conditions affect the rhamnolipid yield dramatically. Purification of rhamnolipid is a complicated step because of the complexity of the fermentation products consisting of proteins, lipids, small molecules and others. Extensive purification steps are required to obtain pure product, which not only require time but also additional operation and reagent expenses. Furthermore, other than optimizing the fermentation and purification strategy, choosing a competent strain for fermentation is also a critical element to reduce production cost because the yields of rhamnolipids vary from species to species.

## Strategies to reduce production cost

**Fig. 3:**
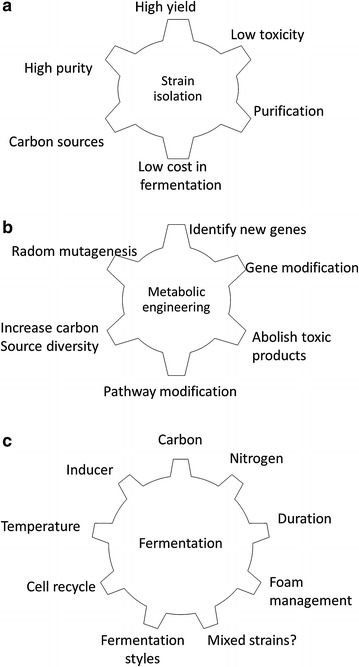
Strategies to reduce rhamnolipid production cost. **a** Strain screening. A good strain can reduce the production cost effectively; **b** strain modification by mutagenesis and metabolic engineering; **c** fermentation. Fermentation is an important step which requires a lot of efforts to optimize. The possible factors that affect fermentation cost are listed

One strategy to reduce the overall rhamnolipid production cost is to lower fermentation (including selection of a suitable strain and an optimized fermentation process) and purification cost (Fig. 3). For example, economic carbon sources such as wastes and renewable substrates [3] can be used to replace some conventional substrates. These include mannitol [33], glucose, glycerol, *n*-paraffin [34, 35], *n*-alkane, polycyclic aromatic hydrocarbons [36] and vegetable oils [37]. The diversity of renewable resources that can be used as carbon and nitrogen sources for rhamnolipid fermentation with reduced substrate cost are discussed in detail in several recent reviews [3, 38–40]. The rhamnolipid yield is largely dependent on the choice of the rhamnolipid producer, the medium composition and the fermentation conditions [32] as the titers of rhamnolipids ranged from 3.9 to 78.56 g/l depending on the cultivation strategies proposed in the literature. The highest titer ever reported was 112 g/l achieved from *P. aeruginosa* fermentation using soybean oil as carbon source in N-limited and Ca^2+^-free medium at 30 °C, pH 6.3 [31, 41, 42].

Downstream processing in many biotechnological processes can contribute up to 50–80% of the total production cost [26, 43] depending on the difficulty of product recovery, which is based on the ionic charge, water solubility and location of the product (intracellular, extracellular or bounded to cell membrane). As rhamnolipids are recovered from fermentation broth, a complex mixture containing unfermented substrates, salts, amino acids, proteins and other metabolic products, downstream purification of rhamnolipids is difficult especially for low rhamnolipid titers [44]. Despite such complexities, it is still possible to purify rhamnolipids with relatively high purity using various proposed strategies such as foam fractionation [44]. The product purification cost can be significantly lowered if (1) cell-free fermentation broth to be used in place of purified rhamnolipids for industrial applications such as in oil recovery or (2) direct usage of rhamnolipid-producing microorganisms applications such as cleaning of oil contamination and additive for animal feeds.

## Application of various strategies to increase rhamnolipid yield

Obtaining a strain with high rhamnolipid yield is the most important step to save production cost, as less complicated purification procedures will be needed. The most commonly used strategy to obtain good strains is to isolate them from the environments where biosurfactant-producing strains can be enriched. Extensive screening has been carried out by many laboratories to isolate and identify various rhamnolipid-producing strains (Table 2). It has been noted that the rhamnolipid yields of *P. aeruginosa* strains remain the highest among the bacteria studied. There are also extensive studies identifying the genes that are critical for rhamnolipid production such that metabolic engineering approach can be used to obtain potential yet safe rhamnolipid-producing strains.

### Improving rhamnolipid yield through random mutagenesis

Mutagenesis through chemical or radiation treatment can be used to improve rhamnolipid yield. For example, a *P. aeruginosa* EBN-8 mutant was obtained by mutagenesis using gamma radiation and was shown to have a better growth on oil refinery wastes than the wild type by producing 8.5 g/l rhamnolipids [45]. *P. fluorescens* 29L obtained under UV mutagenesis was able to produce mutants that were capable of growing on pyrene [46]. Mutagenesis of *P. aeruginosa* by using chemical mutagen *N*-methyl-*N*’-nitro-*N*-nitrosoguanidine (MNNG) generated a mutant which produced 70–120 g/l rhamnolipids [42]. Another study employed UV mutagenesis on rhamnolipid producers from the soil samples generated a mutant with two-fold increment in rhamnolipid production over the parent strain [4]. The random mutagenesis method has been widely used to improve microbial production, but the mutants may lose its productivity after some time.

### Metabolic engineering for non-pathogenic strains

Rhamnolipid biosynthesis in *P. aeruginosa* requires the precursors HAA and dTDP-l-rhamnose, where HAA is synthesized from β-hydroxydecanoyl-ACP. As the biosynthesis pathway for both β-hydroxydecanoyl-ACP and dTDP-l-rhamnose are present in many bacteria, it is therefore possible to construct non-pathogenic recombinant strains by introducing the genes important for rhamnolipid biosynthesis through metabolic engineering tools. HAA is produced in the presence of RhlA, whereas introduction of *rhlB* into the recombinant strain produces mono-rhamnolipid. The heterologous expression of *rhlA*, *rhlB* and *rhlC* will allow the host strain to produce both mono- and di-rhamnolipids. Recombinant strains can produce predominantly di-rhamnolipid if more copies of *rhlC* are expressed in the host strains.

It had been shown that a simple increment in the copy number of *rhlAB* under the control of indigenous strong promoter of *oprL* in *P. aeruginosa* SG could raise the rhamnolipid titer to 20.98 g/l [47]. Similarly, the overexpression of *rhlAB* under *tac* promoter in *B. kururiensis* increased the rhamnolipid titer from 0.78 to 5.76 g/l [48]. The recombinant *B. kururiensis* produces more than 50 rhamnolipid homologues which are typical of both *Pseudomonas* sp. and *Burkholderia* sp., implying that the metabolic engineering can also provide a means in modifying the composition and types of rhamnolipid congeners produced to alter the physicochemical properties of the rhamnolipids [48]. Likewise, *P. chlororaphis,* which was previously reported to produce only mono-rhamnolipids, was shown to be able to produce di-rhamnolipids by overexpressing *rhlC* under a constitutive promoter [49]. The rhamnolipid composition was also altered by site-directed mutagenesis on *rhlB* that resulted in the modification to the volume of the substrate binding pocket [50]. The *rhlAB* operon was also introduced into several other non-pathogenic bacteria, some of which were unable to produce rhamnolipids naturally, such as *E. coli* [50–52], *P. putida* [53, 54], *P. stutzeri* [55] and *P. fluorescens* [54], to enhance rhamnolipid yields. Interestingly, Cabrera-Valladares et al. [52] reported that co-expression of *rmlBDAC* with *rhlAB* was required for rhamnolipid production since dTDP-L-rhamnose was limited in *E. coli*, but the later study suggested that *rmlBDAC* might not be required for rhamnolipid production [51]. The overexpression of *rhlAB* and *rhlRI* from *P. aeruginosa* EMS1 in *P. putida* KCTC produced 7.3 g/l of rhamnolipids [56]. *P. putida* KT2440, capable of growing at high concentrations of rhamnolipids, is reported to be a safe strain for microbial production. By introducing *rhlAB* from *P. aeruginosa,* the recombinant *P. putida* KT2440 was able to produce mono-rhamnolipid. Further deletion of the polyhydroxyalkanoate formation related gene, *phaC,* in *P. putida,* aided in an improvement of the rhamnolipid yield to 1.5 g/g glucose [53]. By replacing the native promoter of *rhlAB* with a synthetic promoter on plasmid pSynPro8oT-rhlAB, heterologous production in recombinant *P. putida* KT2440 using fed-batch fermentation could reach 14.9 g/l when combined with two-phase glucose feeding strategy [57]. All these studies clearly demonstrate the possibility of producing rhamnolipids using non-pathogenic strains.

### Metabolic engineering of *P. aeruginosa* to increase yield

A metabolic engineering approach to enhance rhamnolipid yield in *P. aeruginosa* is either through introducing genes that promote rhamnolipid production or deleting genes that inhibit rhamnolipid biosynthesis. The regulation of rhamnolipid biosynthesis is complicated, further increasing the difficulty to improve rhamnolipid yield via metabolic engineering approaches. Catabolite repression control (Crc) protein is able to activate QS to affect rhamnolipid production through down-regulation of Lon protease. This protease can degrade RhlI protein both in vivo and in vitro. Deletion of the *crc* gene reduces the production of rhamnolipids in *P. aeruginosa*. On the other hand, deletion of *clpX* or *lon* can increase the production of rhamnolipids as they are negative regulators of QS in *P. aeruginosa* [58]. The outer membrane protein EstA affects the production of rhamnolipid in *P. aeruginosa* through its esterase domain. Overexpression of EstA in *P. aeruginosa* results in higher rhamnolipid production than the wild type strain [59]. Hemoglobin from *Vitreoscilla* is useful for increasing oxygen transfer in bacteria, and expression of this gene in *P. aeruginosa* NRRL B-771 increased the titer of rhamnolipids to 8.4 or 13.3 g/L depending on the fermentation media used [60]. Additionally, metabolic engineering can be employed to widen the range of substrates that can be utilized by host microorganisms. For instance, *lacZY* from *E. coli* was cloned into *P. aeruginosa* PAO1 and PG201, enabling the recombinant strains to be able to utilize lactose present in whey waste [61].

### Synthesis of rhamnolipids in vitro

Experimental evidence has shown that purified RhlA is able to catalyze synthesis of HAA in vitro [62]. Both RhlA and RhlB can be purified from *E. coli* [63], making it possible to synthesize rhamnolipids in vitro when both the precursors and enzymes are present. Although some concerns such as catalysis efficiency still remain, it is a simple and promising method to obtain rhamnolipids. Other advantages of in vitro rhamnolipid synthesis include easier downstream purification procedures, as well as elimination of pathogenic effect by obtaining the precursors HAA and dTDP-l-rhamnose from non-pathogenic strains. It is worthwhile to spend efforts on in vitro rhamnolipid synthesis as both mono- and di-rhamnolipids can be obtained using this strategy. However, the expression and purification of RhlC may be challenging using *E. coli* as the host strain [63]. Nevertheless, non-purified RhlC may still be used for in vitro rhamnolipid synthesis.

### Process optimization

Several studies had focused on exploring various fermentation strategies for enhancing rhamnolipid yield by optimization of the fermentation parameters. Currently, many of the proposed methods target on optimization of the components in the growth medium using optimization techniques such as response surface methodology [64]. However, fermentation parameters including the type and feeding profile of substrates, pH, temperature, aeration rate, dissolved oxygen, cell density and availability of in situ product removal, are essential prerequisites for setting up efficient fermentation strategy as these parameters can significantly impact on the rhamnolipid yield [29, 65]. For instance, the use of water-insoluble carbon sources such as palm oil and diesel generally produce rhamnolipids in higher titers as compared to water-soluble carbon sources (e.g. glucose) [29, 66]. The feeding profile of substrates, especially the carbon source, is also an important parameter as fed-batch cultivation is found to be the more effective than batch cultivation to obtain high rhamnolipid titers [57, 65–67]. The key to fed-batch cultivation is to control the substrate concentration at a minimal level that allows optimal microbial growth without catabolite repression or substrate inhibition, and most substrates are utilized for rhamnolipid formation rather than biomass formation. Therefore, kinetic models for substrate utilization, product formation and microbial growth will be useful in developing fed-batch fermentation strategies to dramatically improve the rhamnolipid yield, as evident in several studies [64, 67, 68]. Controlling pH during fermentation also helps in achieving higher rhamnolipid production. Neutral or slightly alkaline pH (pH 7–7.5) allows optimal microbial growth during initial phase of fermentation, after which slightly acidic pH (pH 6–6.5) maximizes rhamnolipid production by suppressing cell growth in middle and late stages of fermentation. In a study conducted by Zhu et al. [65], the rhamnolipid yield was increased from 24.06 to 28.8 g/l simply by controlling the pH during batch fermentation, which was further increased to 70.56 g/l using pH-controlled fed-batch fermentation. The aeration rate to control dissolved oxygen level is also an important fermentation parameter since higher rhamnolipid production is attained with higher dissolved oxygen level [60, 65]. Furthermore, when bioreactors are coupled with tandem defoaming system, in situ product removal helps to prevent product inhibition to enable higher rhamnolipid titers to be reached with other benefits such as obtaining rhamnolipid of high purity with lower purification cost and prevent overflowing of microbial cultures during fermentation [29, 68].

## Reducing the pathogenic impact of *P. aeruginosa* through metabolic engineering

The use of *P. aeruginosa* in rhamnolipid production might be restricted due to its potential pathogenicity, although it is still the most important strain to many researchers and industries because of its high yield of rhamnolipid production. It would be very useful if the pathogenicity of this strain can be diminished through metabolic engineering approach. Pyocyanin is one of the secondary metabolites of *P. aeruginosa*. It is a blue redox-active compound and interferes with multiple cellular functions of human beings [69]. Accumulated studies have shown that pyocyanin, which structure makes it easy to penetrate the cell membrane, is critical for *P. aeruginosa* infection [70]. Synthesis of pyocyanin is through complex cascade of reactions involving several genes such as *phzABCDEFG* and *phzHMS*. The synthesis of pyocyanin is also regulated by the QS systems [71]. Inhibition of pyocyanin biosynthesis is considered as a strategy to rid its toxic impact [70], as well as inhibiting the synthesis of its precursors from the shikimic acid biosynthetic pathway to prevent the expression of the two *phz* operons.

A recent study had characterized a pyocyanin demethylase (PodA) that can oxidize the methyl group of pyocyanin to formaldehyde [72]. PodA is also able to reduce the pyrazine ring through an unusual tautomerizing demethylation reaction, generating products that may be harmless to human cells. It has been shown that treatment of pyocyanin with PodA is able to disrupt *P. aeruginosa* biofilm formation—the critical step for pathogenesis of *P. aeruginosa*. Such a study provides insight into removing the toxic effect pyocyanin through an enzymatic reaction. Introduction of such enzyme into *P. aeruginosa* might provide an efficient way to eradicate the pathogenicity of *P. aeruginosa* where the recombinant strains will have a great potential in industrial applications. Moreover, purified PodA might be used in *P. aeruginosa* fermentation to remove pyocyanin from the broth. Further studies on this topic will be useful to expand the applications of rhamnolipids produced by *P. aeruginosa* to other areas where pathogenicity of *P. aeruginosa* is a concern.

## Concluding remarks

An excellent rhamnolipid-producing strain is critical for cost-effective production of rhamnolipids. Therefore, various efforts have been making to obtain non-pathogenic strains with high rhamnolipid productivity, which include the isolation of potential rhamnolipid-producing strains from the environment, optimization of fermentation conditions and strain improvement by various mutagenesis and metabolic engineering strategies. Metabolic pathway engineering has become an important strategy in this field with the accumulation of knowledge on rhamnolipid biosynthesis and pathogenesis in *P. aeruginosa*. Metabolic engineering can be applied either for reducing the pathogenicity of *P. aeruginosa* or for constructing excellent non-pathogenic rhamnolipid producers, which are two crucial features for economical and safe production of rhamnolipids. Although the cost of product purification is still high, rhamnolipids in high purity can be obtained at an acceptable cost with carefully-designed strategies for various applications especially for those with high-value products such as cosmetics and medicines.

## References

[1] Vatsa P, Sanchez L, Clement C, Baillieul F, Dorey S (2010). Rhamnolipid biosurfactants as new players in animal and plant defense against microbes. Int J Mol Sci.

[2] Paulino BN, Pessoa MG, Mano MCR, Molina G, Neri-Numa IA, Pastore GM (2016). Current status in biotechnological production and applications of glycolipid biosurfactants. Appl Microbiol Biotechnol.

[3] Gudina EJ, Rodrigues AI, de Freitas V, Azevedo Z, Teixeira JA, Rodrigues LR (2016). Valorization of agro-industrial wastes towards the production of rhamnolipids. Biores Technol.

[4] Dobler L, Vilela LF, Almeida RV, Neves BC (2016). Rhamnolipids in perspective: gene regulatory pathways, metabolic engineering, production and technological forecasting. New Biotechnol.

[5] Muller MM, Kugler JH, Henkel M, Gerlitzki M, Hormann B, Pohnlein M, Syldatk C, Hausmann R (2012). Rhamnolipids-next generation surfactants?. J Biotechnol.

[6] Dusane DH, Zinjarde SS, Venugopalan VP, McLean RJC, Weber MM, Rahman P. Quorum sensing: implications on Rhamnolipid biosurfactant production. Biotechnol Genetic Eng Rev. Harding SE editor. 2010;27:159–184. 10.1080/02648725.2010.10648149 21415897

[7] Nitschke M, Costa S (2007). Biosurfactants in food industry. Trends Food Sci Technol.

[8] Abdel-Mawgoud AM, Lepine F, Deziel E (2010). Rhamnolipids: diversity of structures, microbial origins and roles. Appl Microbiol Biotechnol.

[9] Hoskova M, Jezdik R, Schreiberova O, Chudoba J, Sir M, Cejkova A, Masak J, Jirku V, Rezanka T (2015). Structural and physiochemical characterization of rhamnolipids produced by *Acinetobacter calcoaceticus*, *Enterobacter asburiae* and *Pseudomonas aeruginosa* in single strain and mixed cultures. J Biotechnol.

[10] Nitschke M, Costa S, Contiero J (2005). Rhamnolipid surfactants: an update on the general aspects of these remarkable biomolecules. Biotechnol Prog.

[11] Lawniczak L, Marecik R, Chrzanowski L (2013). Contributions of biosurfactants to natural or induced bioremediation. Appl Microbiol Biotechnol.

[12] Hoskova M, Schreiberova O, Jezdik R, Chudoba J, Masak J, Sigler K, Rezanka T (2013). Characterization of rhamnolipids produced by non-pathogenic *Acinetobacter* and *Enterobacter bacteria*. Biores Technol.

[13] Irorere VU, Tripathi L, Marchant R, McClean S, Banat IM (2017). Microbial rhamnolipid production: a critical re-evaluation of published data and suggested future publication criteria. Appl Microbiol Biotechnol.

[14] Toribio J, Escalante AE, Soberón-Chávez G (2010). Rhamnolipids: production in bacteria other than *Pseudomonas aeruginosa*. Eur J Lipid Sci Technol.

[15] Chakrabarty AM (1985). Genetically-manipulated microorganisms and their products in the oil service industries. Trends Biotechnol.

[16] Abdel-Mawgoud AM, Lepine F, Deziel E (2014). A stereospecific pathway diverts beta-oxidation intermediates to the biosynthesis of rhamnolipid biosurfactants. Chem Biol.

[17] Muller MM, Hausmann R (2011). Regulatory and metabolic network of rhamnolipid biosynthesis: traditional and advanced engineering towards biotechnological production. Appl Microbiol Biotechnol.

[18] Lovaglio RB, Silva VL, Ferreira H, Hausmann R, Contiero J (2015). Rhamnolipids know-how: looking for strategies for its industrial dissemination. Biotechnol Adv.

[19] Williams P, Camara M (2009). Quorum sensing and environmental adaptation in *Pseudomonas aeruginosa*: a tale of regulatory networks and multifunctional signal molecules. Curr Opin Microbiol.

[20] Ochsner UA, Reiser J (1995). Autoinducer-mediated regulation of rhamnolipid biosurfactant synthesis in *Pseudomonas aeruginosa*. Proc Natl Acad Sci USA.

[21] Medina G, Juarez K, Valderrama B, Soberon-Chavez G (2003). Mechanism of *Pseudomonas aeruginosa* RhlR transcriptional regulation of the *rhlAB* promoter. J Bacteriol.

[22] Dekimpe V, Deziel E (2009). Revisiting the quorum-sensing hierarchy in *Pseudomonas aeruginosa*: the transcriptional regulator RhlR regulates LasR-specific factors. Microbiol Sgm.

[23] Medina G, Juárez K, Soberón-Chávez G (2003). The *Pseudomonas aeruginosa* rhlAB operon is not expressed during the logarithmic phase of growth even in the presence of its activator RhlR and the autoinducer *N*-butyryl-homoserine lactone. J Bacteriol.

[24] dos Santos AS, Pereira N, Freire DMG (2016). Strategies for improved rhamnolipid production by *Pseudomonas aeruginosa* PA1. Peerj.

[25] Matvyeyeva OL, Vasylchenko OA, Aliieva OR (2014). Microbial biosurfactants role in oil products biodegradation. Int J Environ Bioremed Biodegrad.

[26] Desai JD, Banat IM (1997). Microbial production of surfactants and their commercial potential. Microbiol Mol Biol Rev.

[27] Olvera C, Goldberg JB, Sanchez R, Soberon-Chavez G (1999). The *Pseudomonas aeruginosa* algC gene product participates in rhamnolipid biosynthesis. FEMS Microbiol Lett.

[28] Henkel M, Geissler M, Weggenmann F, Hausmann R. Production of microbial biosurfactants: Status quo of rhamnolipid and surfactin towards large-scale production. Biotechnol J. 2017;12. doi:10.1002/biot.201600561. 10.1002/biot.201600561 28544628

[29] Gong ZJ, Peng YF, Wang QH (2015). Rhamnolipid production, characterization and fermentation scale-up by *Pseudomonas aeruginosa* with plant oils. Biotech Lett.

[30] Lotfabad TB, Ebadipour N, RoostaAzad R (2016). Evaluation of a recycling bioreactor for biosurfactant production by *Pseudomonas aeruginosa* MR01 using soybean oil waste. J Chem Technol Biotechnol.

[31] Lang S, Wullbrandt D (1999). Rhamnose lipids—biosynthesis, microbial production and application potential. Appl Microbiol Biotechnol.

[32] Kaskatepe B, Yildiz S, Gumustas M, Ozkan SA (2015). Biosurfactant production by *Pseudomonas aeruginosa* in kefir and fish meal. Braz J Microbiol.

[33] Nalini S, Parthasarathi R (2013). Biosurfactant production by *Serratia rubidaea* SNAU02 isolated from hydrocarbon contaminated soil and its physico-chemical characterization. Biores Technol.

[34] Sakthipriya N, Doble M, Sangwai JS (2015). Biosurfactant from *Pseudomonas* species with waxes as carbon source—their production, modeling and properties. J Ind Eng Chem.

[35] Sharma D, Ansari MJ, Al-Ghamdi A, Adgaba N, Khan KA, Pruthi V, Al-Waili N (2015). Biosurfactant production by *Pseudomonas aeruginosa* DSVP20 isolated from petroleum hydrocarbon-contaminated soil and its physicochemical characterization. Environ Sci Pollut Res.

[36] Santos AS, Sampaio APW, Vasquez GS, Santa Anna LM, Pereira N, Freire DMG (2002). Evaluation of different carbon and nitrogen sources in production of rhamnolipids by a strain of *Pseudomonas aeruginosa*. Appl Biochem Biotechnol.

[37] Robert M, Mercade ME, Bosch MP, Parra JL, Espuny MJ, Manresa MA, Guinea J (1989). Effect of the carbon source on biosurfactant production by *Pseudomonas aeruginosa* 44T1. Biotech Lett.

[38] Li Q (2017). Rhamnolipid synthesis and production with diverse resources. Front Chem Sci Eng.

[39] Henkel M, Muller MM, Kugler JH, Lovaglio RB, Contiero J, Syldatk C, Hausmann R (2012). Rhamnolipids as biosurfactants from renewable resources: concepts for next-generation rhamnolipid production. Process Biochem.

[40] Merchant R, Banat IM (2012). Microbial biosurfactants: challenges and opportunities for future exploitation. Trends Biotechnol.

[41] Ma KY, Sun MY, Dong W, He CQ, Chen FL, Ma YL (2016). Effects of nutrition optimization strategy on rhamnolipid production in a *Pseudomonas aeruginosa* strain DN1 for bioremediation of crude oil. Biocatal Agric Biotechnol.

[42] Giani C, Wullbrandt D, Rothert R, Meiwes J. *Pseudomonas aeruginosa* and its use in a process for the biotechnological preparation of l-rhamnose. Google Patents; 1996.

[43] Mnif I, Ghribia D (2016). Glycolipid biosurfactants: main properties and potential applications in agriculture and food industry. J Sci Food Agric.

[44] Beuker J, Steier A, Wittgens A, Rosenau F, Henkel M, Hausmann R (2016). Integrated foam fractionation for heterologous rhamnolipid production with recombinant *Pseudomonas putida* in a bioreactor. Amb Express.

[45] Raza ZA, Rehman A, Khan MS, Khalid ZM (2007). Improved production of biosurfactant by a *Pseudomonas aeruginosa* mutant using vegetable oil refinery wastes. Biodegradation.

[46] Husain S (2008). Effect of surfactants on pyrene degradation by *Pseudomonas fluorescens* 29L. World J Microbiol Biotechnol.

[47] Zhao F, Cui QF, Han SQ, Dong HP, Zhang J, Ma F, Zhang Y (2015). Enhanced rhamnolipid production of *Pseudomonas aeruginosa* SG by increasing copy number of *rhlAB* genes with modified promoter. Rsc Adv.

[48] Tavares LFD, Silva PM, Junqueira M, Mariano DCO, Nogueira FCS, Domont GB, Freire DMG, Neves BC (2013). Characterization of rhamnolipids produced by wild-type and engineered *Burkholderia kururiensis*. Appl Microbiol Biotechnol.

[49] Solaiman DKY, Ashby RD, Gunther NW, Zerkowski JA (2015). Dirhamnose-lipid production by recombinant nonpathogenic bacterium *Pseudomonas chlororaphis*. Appl Microbiol Biotechnol.

[50] Han L, Liu P, Peng Y, Lin J, Wang Q, Ma Y (2014). Engineering the biosynthesis of novel rhamnolipids in *Escherichia coli* for enhanced oil recovery. J Appl Microbiol.

[51] Wang QH, Fang XD, Bai BJ, Liang XL, Shuler PJ, Goddard WA, Tang Y (2007). Engineering bacteria for production of rhamnolipid as an agent for enhanced oil recovery. Biotechnol Bioeng.

[52] Cabrera-Valladares N, Richardson AP, Olvera C, Trevino LG, Deziel E, Lepine F, Soberon-Chavez G (2006). Monorhamnolipids and 3-(3-hydroxyalkanoyloxy)alkanoic acids (HAAs) production using *Escherichia coli* as a heterologous host. Appl Microbiol Biotechnol.

[53] Wittgens A, Tiso T, Arndt TT, Wenk P, Hemmerich J, Muller C, Wichmann R, Kupper B, Zwick M, Wilhelm S (2011). Growth independent rhamnolipid production from glucose using the non-pathogenic *Pseudomonas putida* KT2440. Microb Cell Fact.

[54] Ochsner UA, Reiser J, Fiechter A, Witholt B (1995). Production of *Pseudomonas aeruginosa* rhamnolipid biosurfactants in heterologous hosts. Appl Environ Microbiol.

[55] Zhao F, Mandlaa M, Hao J, Liang X, Shi R, Han S, Zhang Y (2014). Optimization of culture medium for anaerobic production of rhamnolipid by recombinant *Pseudomonas stutzeri* Rhl for microbial enhanced oil recovery. Lett Appl Microbiol.

[56] Junjhon J, Lausumpao M, Supasa S, Noisakran S, Songjaeng A, Saraithong P, Chaichoun K, Utaipat U, Keelapang P, Kanjanahaluethai A (2008). Differential modulation of prM cleavage, extracellular particle distribution, and virus infectivity by conserved residues at nonfurin consensus positions of the dengue virus pr-M junction. J Virol.

[57] Beuker J, Barth T, Steier A, Wittgens A, Rosenau F, Henkel M, Hausmann R (2016). High titer heterologous rhamnolipid production. AMB Express.

[58] Yang NN, Ding ST, Chen FF, Zhang X, Xia YJ, Di HX, Cao Q, Deng X, Wu M, Wong CCL (2015). The Crc protein participates in down-regulation of the Lon gene to promote rhamnolipid production and *rhl* quorum sensing in *Pseudomonas aeruginosa*. Mol Microbiol.

[59] Wilhelm S, Gdynia A, Tielen P, Rosenau F, Jaeger KE (2007). The autotransporter esterase EstA of *Pseudomonas aeruginosa* is required for rhamnolipid production, cell motility, and biofilm formation. J Bacteriol.

[60] Kahraman H, Erenler SO (2012). Rhamnolipid production by *Pseudomonas aeruginosa* engineered with the *Vitreoscilla* hemoglobin gene. Appl Biochem Microbiol.

[61] Koch AK, Reiser J, Kappeli O, Fiechter A (1988). Genetic construction of lactose-utilizing strains of *Pseudomonas aeruginosa* and their application in biosurfactant production. Bio Technology.

[62] Zhu K, Rock CO (2008). RhlA converts beta-hydroxyacyl-acyl carrier protein intermediates in fatty acid synthesis to the beta-hydroxydecanoyl-beta-hydroxydecanoate component of rhamnolipids in *Pseudomonas aeruginosa*. J Bacteriol.

[63] Kiss K, Ng WT, Li Q (2017). Production of rhamnolipids-producing enzymes of *Pseudomonas* in *E. coli* and structural characterization. Front Chem Sci Eng.

[64] Henkel M, Schmidberger A, Vogelbacher M, Kühnert C, Beuker J, Bernard T, Schwartz T, Syldatk C, Hausmann R (2014). Kinetic modeling of rhamnolipid production by *Pseudomonas aeruginosa* PAO1 including cell density-dependent regulation. Appl Microbiol Biotechnol.

[65] Zhu LQ, Yang X, Xue CY, Chen Y, Qu L, Lu WY (2012). Enhanced rhamnolipids production by *Pseudomonas aeruginosa* based on a pH stage-controlled fed-batch fermentation process. Biores Technol.

[66] Md Noh NA, Mohd Salleh S, Yahya ARM (2014). Enhanced rhamnolipid production by *Pseudomonas aeruginosa* USM-AR2 via fed-batch cultivation based on maximum substrate uptake rate. Lett Appl Microbiol.

[67] Satya Eswari J, kavya K (2016). Optimal feed profile for the rhamnolipid kinetic models by using Tabu search: metabolic view point. AMB Express.

[68] Medina-Moreno SA, Jiménez-Islas D, Gracida-Rodríguez JN, Gutiérrez-Rojas M, Díaz-Ramírez IJ (2011). Modeling rhamnolipids production by *Pseudomonas aeruginosa* from immiscible carbon source in a batch system. Int J Environ Sci Technol.

[69] Ran H, Hassett DJ, Lau GW (2003). Human targets of *Pseudomonas aeruginosa* pyocyanin. Proc Natl Acad Sci.

[70] Lau GW, Hassett DJ, Ran H, Kong F (2004). The role of pyocyanin in *Pseudomonas aeruginosa* infection. Trends Mol Med.

[71] Mavrodi DV, Bonsall RF, Delaney SM, Soule MJ, Phillips G, Thomashow LS (2001). Functional analysis of genes for biosynthesis of pyocyanin and phenazine-1-carboxamide from *Pseudomonas aeruginosa* PAO1. J Bacteriol.

[72] Costa KC, Glasser NR, Conway SJ, Newman DK (2017). Pyocyanin degradation by a tautomerizing demethylase inhibits <em>*Pseudomonas aeruginosa*</em> biofilms. Science.

[73] Lan GH, Fan Q, Liu YQ, Liu Y, Liu YC, Yin XB, Luo M (2015). Effects of the addition of waste cooking oil on heavy crude oil biodegradation and microbial enhanced oil recovery using *Pseudomonas* sp. SWP-4. Biochem Eng J.

[74] Li Q, Kang C, Wang H, Liu C, Zhang C (2002). Application of microbial enhanced oil recovery technique to Daqing Oilfield. Biochem Eng J.

[75] Bertrand JC, Bonin P, Goutx M, Gauthier M, Mille G (1994). The potential application of biosurfactants in combating hydrocarbon pollution in marine environments. Res Microbiol.

[76] Bragg JR, Prince RC, Harner EJ, Atlas RM (1994). Effectiveness of bioremediation for the Exxon-Valdez oil-spill. Nature.

[77] Anna LMS, Soriano AU, Gomes AC, Menezes EP, Gutarra MLE, Freire DMG, Pereira N (2007). Use of biosurfactant in the removal of oil from contaminated sandy soil. J Chem Technol Biotechnol.

[78] Roy S, Chandni S, Das I, Karthik L, Kumar G, Rao KVB (2015). Aquatic model for engine oil degradation by rhamnolipid producing *Nocardiopsis* VITSISB. 3 Biotech.

[79] Sachdev DP, Cameotra SS (2013). Biosurfactants in agriculture. Appl Microbiol Biotechnol.

[80] Kim SK, Kim YC, Lee S, Kim JC, Yun MY, Kim IS (2011). Insecticidal activity of rhamnolipid isolated from *Pseudomonas* sp. EP-3 against green peach aphid (*Myzus persicae*). J Agric Food Chem.

[81] Deepika KV, Sridhar PR, Bramhachari PV (2015). Characterization and antifungal properties of rhamnolipids produced by mangrove sediment bacterium *Pseudomonas aeruginosa* strain KVD-HM52. Biocatal Agric Biotechnol.

[82] Sha RY, Jiang LF, Meng Q, Zhang GL, Song ZR (2012). Producing cell-free culture broth of rhamnolipids as a cost-effective fungicide against plant pathogens. J Basic Microbiol.

[83] Benincasa M, Abalos A, Oliveira I, Manresa A (2004). Chemical structure, surface properties and biological activities of the biosurfactant produced by *Pseudomonas aeruginosa* LBI from soapstock. Antonie Van Leeuwenhoek.

[84] Hultberg M, Bergstrand KJ, Khalil S, Alsanius B (2008). Characterization of biosurfactant-producing strains of fluorescent pseudomonads in a soilless cultivation system. Antonie Van Leeuwenhoek.

[85] Magalhaes L, Nitschke M (2013). Antimicrobial activity of rhamnolipids against *Listeria monocytogenes* and their synergistic interaction with nisin. Food Control.

[86] Irie Y, O’Toole GA, Yuk MH (2005). *Pseudomonas aeruginosa* rhamnolipids disperse *Bordetella bronchiseptica* biofilms. FEMS Microbiol Lett.

[87] Trummler K, Effenberger F, Syldatk C (2003). An integrated microbial/enzymatic process for production of rhamnolipids and l-(+)-rhamnose from rapeseed oil with *Pseudomonas* sp. DSM 2874. Eur J Lipid Sci Technol.

[88] Schooling SR, Charaf UK, Allison DG, Gilbert P (2004). A role for rhamnolipid in biofilm dispersion. Biofilms.

[89] Diaz De Rienzo MA, Stevenson PS, Marchant R, Banat IM (2016). Effect of biosurfactants on *Pseudomonas aeruginosa* and *Staphylococcus aureus* biofilms in a BioFlux channel. Appl Microbiol Biotechnol.

[90] Andersen KK, Otzen DE (2014). Folding of outer membrane protein A in the anionic biosurfactant rhamnolipid. FEBS Lett.

[91] Zheng T, Xu YS, Yong XY, Li B, Yin D, Cheng QW, Yuan HR, Yong YC (2015). Endogenously enhanced biosurfactant production promotes electricity generation from microbial fuel cells. Biores Technol.

[92] Kiran GS, Ninawe AS, Lipton AN, Pandian V, Selvin J (2016). Rhamnolipid biosurfactants: evolutionary implications, applications and future prospects from untapped marine resource. Crit Rev Biotechnol.

[93] Muller MM, Hormann B, Kugel M, Syldatk C, Hausmann R (2011). Evaluation of rhamnolipid production capacity of *Pseudomonas aeruginosa* PAO1 in comparison to the rhamnolipid over-producer strains DSM 7108 and DSM 2874. Appl Microbiol Biotechnol.

[94] Zhang XS, Xu DJ, Zhu CY, Lundaa T, Scherr KE (2012). Isolation and identification of biosurfactant producing and crude oil degrading *Pseudomonas aeruginosa* strains. Chem Eng J.

[95] Grosso-Becerra MV, Gonzalez-Valdez A, Granados-Martinez MJ, Morales E, Servin-Gonzalez L, Mendez JL, Delgado G, Morales-Espinosa R, Ponce-Soto GY, Cocotl-Yanez M, Soberon-Chavez G (2016). *Pseudomonas aeruginosa* ATCC 9027 is a non-virulent strain suitable for mono-rhamnolipids production. Appl Microbiol Biotechnol.

[96] Gunther NW, Nunez A, Fett W, Solaiman DKY (2005). Production of rhamnolipids by *Pseudomonas chlororaphis*, a nonpathogenic bacterium. Appl Environ Microbiol.

[97] Vasileva-Tonkova E, Galabova D, Stoimenova E, Lalchev Z (2006). Production and properties of biosurfactants from a newly isolated *Pseudomonas fluorescens* HW-6 growing on hexadecane. Zeitschrift Fur Naturforschung C.

[98] Abouseoud M, Yataghene A, Amrane A, Maachi R (2008). Biosurfactant production by free and alginate entrapped cells of *Pseudomonas fluorescens*. J Ind Microbiol Biotechnol.

[99] Bhardwaj G, Cameotra SS, Chopra HK (2015). Utilization of oil industry residues for the production of rhamnolipids by *Pseudomonas indica*. J Surf Deterg.

[100] Onbaslil D, Aslim B (2009). Biosurfactant production in sugar beet molasses by some *Pseudomonas* spp.. J Environ Biol.

[101] Onwosi CO, Odibo FJC (2012). Effects of carbon and nitrogen sources on rhamnolipid biosurfactant production by *Pseudomonas nitroreducens* isolated from soil. World J Microbiol Biotechnol.

[102] Tuleva BK, Ivanov GR, Christova NE (2002). Biosurfactant production by a new P*seudomonas putida* strain. Zeitschrift Fur Naturforschung C.

[103] Janek T, Łukaszewicz M, Krasowska A (2013). Identification and characterization of biosurfactants produced by the Arctic bacterium *Pseudomonas putida* BD2. Coll Surf B.

[104] Singh DN, Tripathi AK (2013). Coal induced production of a rhamnolipid biosurfactant by *Pseudomonas stutzeri*, isolated from the formation water of Jharia coalbed. Biores Technol.

[105] Costa S, Deziel E, Lepine F (2011). Characterization of rhamnolipid production by *Burkholderia glumae*. Lett Appl Microbiol.

[106] Hormann B, Muller MM, Syldatk C, Hausmann R (2010). Rhamnolipid production by *Burkholderia plantarii* DSM 9509(T). Eur J Lipid Sci Technol.

[107] Haussler S, Nimtz M, Domke T, Wray V, Steinmetz I (1998). Purification and characterization of a cytotoxic exolipid of *Burkholderia pseudomallei*. Infect Immun.

[108] Dubeau D, Deziel E, Woods DE, Lepine F (2009). *Burkholderia thailandensis* harbors two identical rhl gene clusters responsible for the biosynthesis of rhamnolipids. BMC Microbiol.

[109] Funston SJ, Tsaousi K, Rudden M, Smyth TJ, Stevenson PS, Marchant R, Banat IM (2016). Characterising rhamnolipid production in *Burkholderia thailandensis* E264, a non-pathogenic producer. Appl Microbiol Biotechnol.

[110] Chen J, Huang PT, Zhang KY, Ding FR (2012). Isolation of biosurfactant producers, optimization and properties of biosurfactant produced by *Acinetobacter* sp. from petroleum-contaminated soil. J Appl Microbiol.

[111] Rooney AP, Price NPJ, Ray KJ, Kuo TM (2009). Isolation and characterization of rhamnolipid-producing bacterial strains from a biodiesel facility. FEMS Microbiol Lett.

[112] Vasileva-Tonkova E, Gesheva V (2005). Glycolipids produced by *Antarctic Nocardioides* sp. during growth on *n*-paraffin. Process Biochem.

[113] Nayak AS, Vijaykumar MH, Karegoudar TB (2009). Characterization of biosurfactant produced by *Pseudoxanthomonas* sp. PNK-04 and its application in bioremediation. Int Biodeterior Biodegrad.

[114] Christova N, Tuleva B, Lalchev Z, Jordanova A, Jordanov B (2004). Rhamnolipid biosurfactants produced by *Renibacterium salmoninarum* 27BN during growth on *n*-hexadecane. Zeitschrift Fur Naturforschung C.

[115] Yan X, Sims J, Wang B, Hamann MT (2014). Marine actinomycete *Streptomyces* sp. ISP2-49E, a new source of Rhamnolipid. Biochem Syst Ecol.

[116] Lee M, Kim MK, Vancanneyt M, Swings J, Kim SH, Kang MS, Lee ST (2005). *Tetragenococcus koreensis* sp. nov., a novel rhamnolipid-producing bacterium. Int J Syst Evol Microbiol.

[117] Rezanka T, Siristova L, Sigler K (2011). Rhamnolipid-producing thermophilic bacteria of species *Thermus* and *Meiothermus*. Extremophiles.

